# Nursing Home Satisfaction Surveys: Differences by Race, Age, and Gender

**DOI:** 10.1093/geroni/igab046.272

**Published:** 2021-12-17

**Authors:** Nicholas Castle, John Harris, David Wolf

**Affiliations:** 1 West Virginia University, Allison Park, Pennsylvania, United States; 2 University of Pittsburgh, Pittsburgh, Pennsylvania, United States; 3 Bellarmine, Louisville, Kentucky, United States

## Abstract

Nursing home satisfaction information has gained substantial traction as a quality indicator representing the consumers perspective. However, very little research has examined differences in satisfaction related to race, age and gender. As a quality metric, satisfaction measures are variously used for quality improvement, benchmarking, public reporting, and for adjustment to payments. As such, valid comparisons among facilities are important. To our knowledge, no adjustment to satisfaction scores are currently used for nursing homes. However, in many other settings this is a common practice. In this research, nursing home resident, family, and discharge satisfaction scores were examined from >4,000 participants. The data were collected in 2020 and come from 420 facilities. Satisfaction information came from the CoreQ surveys, which include 23 individual questions four of which can be combined to produce an overall satisfaction score. These CoreQ nursing home surveys are endorsed by NQF. Generally lower overall satisfaction scores were found for family members compared to current residents or discharged residents. Minorities (Black, Asian, Hispanic) had lower overall satisfaction scores compared to whites; however, the differences were not significant at conventional levels. Participants of the lowest age (<65 years) were significantly (p=<.05) less satisfied than older participants (>75 years) and males were significantly (p=<.05) less satisfied than females. The findings indicate that some case-mix adjustment may be applicable for nursing home satisfaction scores.

